# Lower gastrointestinal bleeding in pregnancy: Differential diagnosis, assessment and management

**DOI:** 10.1177/1753495X20948300

**Published:** 2020-09-01

**Authors:** L Story, S Rafique, N Samadi, J Mawdsley, B Singh, A Banerjee

**Affiliations:** 1 Department of Women and Children’s Health King’s College, London, UK; 2 Women’s Services, Guy’s and St Thomas’ NHS Foundation Trust, London, UK; 3 King’s College London Medical School, London, UK; 4 Department of Gastroenterology, Guy’s and St Thomas’ NHS Foundation Trust, London, UK; 5 Department of Surgery, University Hospitals Leicester, Leicester, UK

**Keywords:** Rectal bleeding, pregnancy, haemorrhoids, anal fissure, cancer, inflammatory bowel disease

## Abstract

Rectal bleeding is a common symptom experienced by pregnant women. Although the majority of cases are attributable to benign conditions such as haemorrhoids and anal fissures, other more serious diagnoses such as inflammatory bowel disease and malignancy should not be overlooked. Most investigations are safe during pregnancy and these should not be withheld as significant implications on both fetal and maternal morbidity may result. In these cases, a multidisciplinary team approach is essential. This review explores the differential diagnosis, investigation and management of rectal bleeding during pregnancy.

## Introduction

Rectal bleeding is a symptom commonly reported by pregnant women. The physiological changes associated with pregnancy and the gravid uterus can exacerbate common benign conditions such as haemorrhoids and anal fissures. It is imperative, however, that alternative diagnoses are not overlooked. Conditions such as inflammatory bowel disease (IBD), if not appropriately managed can be associated with significant adverse pregnancy outcomes, so prompt diagnosis and assessment of disease activity are imperative.^1^ In addition, women are increasingly deferring childbirth and as maternal age is rising, malignancies identified during pregnancy are increasing.^2^ Thorough investigation and management should therefore not be deferred and a multidisciplinary team approach involving obstetricians, radiologists, gastroenterologists and surgeons is imperative.

### Assessment

A thorough history is essential to determine the characteristics of the bleeding and any other concomitant symptoms. Specific features may help discern the cause, including volume, frequency, whether it is fresh blood covering the stool (indicative of more distal pathology such as haemorrhoids) or mixed with the stool (consistent with more proximal pathology). Localised peri-anal symptoms are important as pain during defecation is commonly noted with an anal fissure, whilst haemorrhoidal bleeding is typically described as being painless. It should be noted whether symptom onset occurred after eating certain foods or following travel and whether other close contacts are also unwell with diarrhoea or vomiting, as this may indicate an infective cause.

A history of change in stool frequency or abdominal pain should be elicited, as it is relevant to other causes of rectal bleeding such as IBD or colorectal malignancy. However, some of the ‘red-flag symptoms’,^3^ which may alert to these conditions in the non-pregnant population (see Table 1), including anaemia and alterations in bowel habit, can overlap with symptoms attributable to the physiological changes that occur during pregnancy and other features, such as weight loss, may be more difficult to detect. Enquiring whether these symptoms preceded the pregnancy is therefore imperative.

**Table 1.** Red flag symptoms which may be associated with underlying colorectal malignancy in the non-pregnant population (adapted from NICE).

Unexplained weight loss
Altered bowel habit
Iron deficiency anaemia
Abdominal pain
Family history of bowel cancer

Extra-gastrointestinal problems such as skin, eye or joint symptoms or signs can be suggestive of IBD and a family history is relevant to both colorectal malignancy and IBD. The drug history should enquire as to whether the woman is taking any anticoagulants such as low-molecular-weight heparin or antiplatelet agents such as aspirin, both commonly used during pregnancy. Although not causative, these may exacerbate rectal bleeding.

An examination of the abdominal system should include visual inspection of the perineal area and digital rectal examination, as these may be sufficient to give a diagnosis of haemorrhoids or anal fissure or identify a rectal mass. Abdominal palpation may elicit pain, although masses may be harder to detect in the presence of the gravid uterus.

Basic laboratory tests such as a full blood count (FBC) may reveal features of anaemia indicating significant blood loss. Raised inflammatory markers such as C-reactive protein (CRP) can be useful in diagnosing IBD or monitoring established disease. Levels may also rise due to the inflammatory response associated with diverticulitis or infective diarrhoea. Erythrocyte sedimentation rate (ESR) is not routinely used as pregnancy is associated with physiological rises, making results difficult to interpret.^4^

Stool culture can be useful for excluding infective causes of rectal bleeding and is also relevant to exclude concomitant infection in established (IBD). Faecal calprotectin is a non-specific marker of gastrointestinal (GI) inflammation and so will be raised in inflammatory causes of GI bleeding such as infection, IBD or diverticulitis. It can also be used as a marker of disease activity in established IBD. However, studies assessing the value of calprotectin during pregnancy are conflicting^5–7^ and results therefore need to be interpreted only with correlation within the clinical context. Faecal immunochemical testing (FIT), which detects minute amounts of blood in the stool, is increasingly being used in primary care to screen for bowel cancer. This is an antibody test specific for human haemoglobin, unlike the faecal occult blood test, which has a higher rate of false-positives so is less specific. However, there is little published literature on use of FIT in pregnancy and its value in established rectal bleeding is unclear.

If a woman is referred because of lower GI bleeding, assessment of the anus and rectum can be undertaken using a proctoscope and a rigid sigmoidoscope without the need of any bowel preparation. They have a role in assessing common anorectal causes of bleeding such as haemorrhoids or anal fissures.

Endoscopy in pregnancy should not be withheld if necessary and it can facilitate prompt diagnosis and management of various GI conditions.^8^ Studies have demonstrated the safety of both flexible sigmoidoscopy and colonoscopy in pregnancy,^9,10^ although it is often deferred until the second trimester. Flexible sigmoidoscopy has the advantage of requiring only an enema as preparation and is sufficiently brief that the need for sedation can usually be avoided. It is of particular use when symptoms are suggestive of disease of either the rectum or sigmoid. Specific procedural, anaesthetic and other medication adjustments should be considered to optimise safety in pregnant women^8^ although a single dose of standard sedatives used in endoscopy such as a benzodiazepine or fentanyl is considered unlikely to cause harm.^8^ Doses do not need to be altered but care should be taken to avoid over-sedation and hypotension which may result in foetal hypoxia. Both flexible sigmoidoscopy and colonoscopy should be performed in the left lateral position to avoid vena cava or aortic compression.

In addition to endoscopy, other imaging modalities can be used in pregnancy to aid the diagnosis of Per rectum (PR) bleeding. MRI and ultrasound are used in preference to CT and plain radiography in order to minimise ionising radiation exposure to the foetus.^11^ However, if they are deemed an essential part of the diagnostic workup, these may also be performed.^11^ The foetus is unlikely to suffer adverse effects if cumulative radiation is below 100 mGy (a CT is unlikely to result in an exposure of more than 50 mGy).^12^,^13^ A summary of the differential diagnosis and investigations can be seen in Table 2.

**Table 2.** Differential diagnosis, clinical features and investigations of rectal bleeding in pregnancy.

Differential diagnosis for rectal bleeding in pregnancy	Clinical features	Investigations
Hemorrhoids	*History* Bright red blood coating stool Pruritus ani Pain if prolapsed Exacerbated by constipation or obstructive defecation Mucus discharge *Examination* Rectal examination	Proctoscopy if indicated
Anal fissures	*History* Blood on wiping or may be coating the stool Pain on defecation *Examination* Visual inspection Unlikely to tolerate a rectal examination due to anal spasm	NA
Infective diarrhoea	*History* Loose stool Associated with food or travel Other close contacts may also be affected May be associated with systemic symptoms, abdominal pain and nausea *Examination* Abdominal palpation may elicit pain	FBC, CRP Stool culture
Diverticular disease (complications include diverticulitis, obstruction, fistula, bleeding, abscess/perforation)	*History* Abdominal pain Constipation Symptoms of systemic infection if diverticulitis Symptoms of obstruction if associated with a diverticular structure Symptoms of fistula *Examination* Abdominal exam Acute abdomen if perforation Rectal examination	Can be an incidental finding during endoscopy Raised inflammatory markers if diverticulitis CT may diagnosis diverticulitis, diverticular abscess or perforation
Inflammatory bowel disease	*History* Blood mixed with stools Abdominal pain Diarrhoea Fatigue and systemic malaise Extra-intestinal manifestation such as skin lesions (erythema nodosum or pyoderma gangrenosum), joint pain, eye symptoms Family history *Examination* Abdominal examination, assessment of non-GI manifestations including skin/eye lesions and joint involvement	FBC, CRP Stool cultures, faecal calprotectin Colonoscopy or flexible sigmoidoscopy as indicated Small bowel MRI may be considered if other symptoms suggestive of small bowel disease
Colorectal cancer	*History* Loss of appetite Abdominal pain Alteration in bowel habit Family history *Examination* Rectal examination assessing for masses	FBC, liver function tests, renal function Colonoscopy/flexible sigmoidoscopy/CT colon, Consider pelvic MRI CT scan chest/abdo/pelvis for staging if risks outweigh benefit

CRP: C-reactive protein; FBC: full blood count; GI: gastrointestinal.

### Differential diagnosis and management

#### Haemorrhoids

Protocological conditions such as those associated with haemorrhoids and anal fissures are common in pregnancy.^14^ Approximately 25–35% of pregnant women experience haemorrhoids^15,16^ and in some series this has even been reported to be as high as 75–85%.^17^

The aetiology in pregnancy is related to increasing circulating volume, the effects of progesterone on smooth muscle in the walls of the veins and increased intra-abdominal pressure resulting from the gravid uterus, causing compression of the superior rectal veins.^18^ Haemorrhoids result from distal displacement of the anal cushions, prominences of anal mucosa formed from connective tissue, smooth muscle and both arterial and venous blood vessels.^19^ They can be subcategorised into internal and external, the latter of which do not require treatment unless symptomatic. External haemorrhoids can present as a painful perianal swelling which can be managed by analgesia and usually resolves. It can be distinguished from prolapsed haemorrhoids which usually have a circumferential appearance at the 3, 7 and 11 o’clock position representative of the anal cushions.

Commonly associated symptoms in addition to bright red blood on wiping or coating the stools include anal pain, pruritus ani, mucus discharge, faecal incontinence and constipation.^14^ The diagnosis is confirmed from examining the anus and anal canal, digital examination and proctoscopy if required.

Haemorrhoids can often be managed successfully by increasing dietary fibre and hydration, laxatives and topical analgesia.^18^ Although comprehensive studies are lacking, assessing the efficacy and safety of types of laxatives during pregnancy, they are routinely used due to minimal systemic absorption.^20,21^ Stimulant and osmotic laxatives may rarely be associated with dehydration or electrolyte disturbances and should be used with caution if used over prolonged periods of time. Other medical treatments can be administered including flavonoids which have been shown to be safe in pregnancy.^22,23^

Mechanical interventions can be considered with failure of conservative treatment. Banding^24^ and cryotherapy^24^ are generally only used in patients who have persistent symptoms despite medical management and their use in pregnancy is rare. Surgery may be considered if haemorrhoids are thrombosed, severely prolapsed and incarcerated or ulcerated, associated with significant bleeding or have failed to respond to more conservative measures.^25,26^ Although botulinum toxin has been injected as a treatment for both haemorrhoids and chronic anal fissures it is not recommended during pregnancy. For many women, haemorrhoids will regress and symptoms significantly improve post-partum.^26^

#### Anal fissures

Anal fissures result from a tear in the anal mucosa, often caused by trauma such as a large bowel motion and constipation. Pregnancy again predisposes to anal fissures, due to decreased colonic motility resulting from the vasodilatory effects of prostaglandins and vascular endothelial substances and pressure on the recto sigmoid colon by the gravid uterus. In one study, the incidence in pregnancy was approximately 3%.^27^ Bright red bleeding can result on wiping and is often associated with sharp pain on defecation. The history alone is often sufficient to make a diagnosis but inspection is the most important aspect of the examination, confirming suspicion of the fissure and excluding other pathology. Rectal exam may not be possible due to pain but this should be undertaken if there is uncertainty regarding the diagnosis. Anal fissures may also be found with concomitant haemorrhoids.^27,28^

In the non-pregnant population up to 87% of acute fissures will resolve with conservative management such as a high fibre diet, laxatives^29^ and topical agents. Topical nitric oxide donors (including glyceryl trinitrate, isosorbide dinitrate and isosorbide mononitrate) and calcium channel blockers (nifedipine and diltiazem), both oral and topical, have been assessed in the non-pregnant population with the latter being more effective although not currently licenced for this indication.^30^ However, neither have been evaluated in pregnancy.^31^ Where symptoms do not improve within eight weeks of these conservative therapies, surgery or botulinum toxin injection can be considered in the non-pregnant population^32^ but this is not advocated during pregnancy.^31^ Surgery involves a lateral sphincterotomy and has the disadvantage of permanently dividing the internal sphincter, which can lead to long-term problems with continence.

#### Infective diarrhoea

Some infective pathogens may result in diarrhoea mixed with blood and mucus due to colonic inflammation. The patient may report a history of becoming unwell after eating a certain food and other close contacts may report similar symptoms. A travel history should also be elicited. Causative organisms may include Shigella, Campylobacter, Salmonella and *E. coli* and can be associated with systemic features such as fever. *Clostridium difficile* may also be considered in hospitalised women who have had a recent course of antibiotics. Stool cultures should be sent and, depending on the pathogen identified, antibiotics considered.^33^ A fluid balance assessment should be undertaken as intravenous fluid and electrolyte replacement may additionally be required.

#### Inflammatory bowel disease

IBD consists of ulcerative colitis (UC) and Crohn’s disease (CD). In UC, the inflammation extends proximally from the rectum to a variable degree in the colon and is confined in the mucosal layer. In CD, the inflammation is non-continuous and can occur as discrete skip lesions in any part of the digestive tract from mouth to anus.^34^ The inflammation is trans-mural.

Rectal bleeding can be a feature of both UC and CD, especially if the latter is colonic in distribution, but is more common in UC. Other features often associated include liquid diarrhoea, increased stool frequency, abdominal pain and urgency of defecation. Extra-intestinal manifestations may also be present and these should be explored in the history. Features may include musculoskeletal symptoms (indicating peripheral arthropathy, sacroiliitis or ankylosing spondylitis), eye manifestations (conjunctivitis, iridocyclitis, episcleritis) or skin/mucosal features (aphthous ulcers, erythema nodosum and pyoderma gangrenosum).

Women may present with a new diagnosis of IBD in pregnancy or a relapse of pre-existing disease. An accurate diagnosis and assessment of disease activity are important to allow appropriate management improving outcomes for both the mother and child.^35^ If conception occurs at a time of quiescent disease, the risk of relapse is no more than in non-pregnant women with IBD but is greater in UC than CD.^36^,^37^

Although the majority of women have full-term uncomplicated pregnancies, active disease may impact on pregnancy outcomes, with an increased risk of miscarriage rates, foetal growth restriction and preterm delivery.^38,39^ Active disease also increases maternal risks such as venous thromboembolism,^40^ therefore accurate diagnosis and management of flares are crucial.

When investigating a potential new diagnosis of IBD or managing existing disease FBC, CRP, stool cultures and faecal calprotectin should be performed (see above). Endoscopy may be required if a new diagnosis is suspected or if it remains unclear if bleeding is due to active disease. MRI can be used to assess small bowel CD or right-sided colonic disease. Contrast-enhanced ultrasound is also becoming more prevalent to assess disease activity and the response of patients to therapy^41^; however, its use during pregnancy needs to be further evaluated. CT is usually reserved for when there are concerns about extra-luminal complications of IBD such as perforation or intra-abdominal collections.

The management of IBD falls out of the remit of this article. Appropriate treatments include mesalazine and sulfasalazine which can be safely used in pregnancy although if sulfasalazine is used, additional folic acid should be prescribed, in order to minimise the risks of neural tube defects due to its anti-folate action.^42^ Corticosteroids should not be withheld if required to control acute flares, but are not the optimal long-term treatment.^42^ They are associated with an increased risk of gestational diabetes mellitus, hypertension and infections as well as preterm delivery.^42^ Screening for diabetes, should therefore be undertaken at 28 weeks or sooner if additional risk factors are present. Parenteral steroids may be required during labour if more than 5 mg prednisolone is given for more than three weeks prior to delivery. The immunomodulators azathioprine and mercaptopurine can be used safely in pregnancy^43^ but methotrexate should be avoided.^44^ Anti-TNF agents (Infliximab, adalimumab) are the most commonly used biological drugs in IBD and would appear to be safe in pregnancy with no increased risk. They are certainly considered safer than the risk of ongoing active IBD.^12^

#### Colorectal malignancies/polyps

Although the coexistence of pregnancy and malignancy is rare, with an incidence of approximately 1 in 1000 pregnancies,^45^ as women are increasingly choosing to defer pregnancy, childbirth at advanced maternal age is becoming increasingly frequent.^46,47^ As the incidence of malignancy increases with advancing age, colorectal cancer in pregnant women is only likely to rise in the future.

Although breast, cervical, skin and haematological malignancies are considered to be the most common cancers during pregnancy,^45^ the incidence of colorectal cancer is rising.^48^ One retrospective cohort study by Shim et al., 2016 reported 98 new diagnoses of malignancy during pregnancy in a sample of 50,412 pregnancies over a 19-year-period^49^ and of these cases, GI cancer was the second most common diagnosis accounting for 17% of cases. The majority of these cases arose from the rectum.^50^

Pellino et al. conducted a systematic review of colorectal cancer diagnosed during pregnancy including 79 papers encompassing 119 women^48^ finding that rectal bleeding was the most common presenting complaint occurring in 47% of cases. Other symptoms at presentation included abdominal pain (37.6%), constipation (14.1%), acute bowel obstruction (9.4%) and acute peritonitis secondary to perforation (2.4%). Metastatic disease was found to be present in 48% of cases at the time of initial diagnosis.^48^ Weight loss, tenesmus, nausea and a change in bowel movement have also been reported, however, the latter symptoms are common in pregnancy and diagnosis may be delayed as a result.^51^ A family history of colorectal malignancies should also be sought.

Rectal examination should be performed and endoscopic assessment should also be performed, as in non-pregnant individuals. Rectal examination and sigmoidoscopy have been shown to diagnose more than 80% of colorectal tumours in pregnant women^24^ and biopsies can facilitate a tissue diagnosis. In some circumstances, treatment may be possible for example removal of polyp (benign, premalignant or malignant). Carcinoembryonic antigen (CEA) is a tumour marker used to monitor disease progression in non-pregnant women has little value in the pregnant population as levels are raised by pregnancy itself.^50^ Staging investigations should not be withheld if pregnant.

The management of colorectal cancer in pregnancy is outside the scope of this article. Surgical treatment should be undertaken when indicated, particularly in the first and second trimesters.^30,48,52^ Adjuvant chemotherapy can be considered after the first trimester of pregnancy, however, due to potential adverse effects including myelosuppression and bleeding it is not advised within three weeks of delivery or after 35 weeks’ gestation.^53^ Radiation should be avoided.

#### Diverticular disease

Diverticular disease, where outpouchings or diverticula of the wall of the large intestine occur, is a common condition affecting 5–10% of the population over 45.^54^ Less than 5% of cases are diagnosed before 40,^55^ however, as women are deferring childbirth until later in life, the incidence during pregnancy is likely to rise. Case reports have been published of pregnant women presenting with rectal bleeding being diagnosed with diverticulitis.^56^ Commonly associated features include abdominal pain, nausea and vomiting and febrile symptoms. Acute diverticulitis can either be uncomplicated or complicated. The former is generally treated by antibiotics whereas the latter includes abscess, perforation, fistula, bleeding and obstruction and may require surgery if conservative management fails. Diverticular disease can also be an incidental finding during endoscopy.

Imaging modalities can include CT where absolutely necessary. A FBC and CRP may indicate signs of infection/inflammation.

Antibiotics can be used during pregnancy and, as in the case of complicated diverticulitis reported by Bodner et al., surgical intervention may also be required.^56^

## Conclusion

Rectal bleeding in pregnancy is a common presentation, with a range of differential diagnoses. Haemorrhoids should not be the assumed diagnosis and a thorough history, examination and targeted investigations are essential. Appropriate investigations should not be withheld due to pregnancy.
